# The Effects of Virtual Reality Training on Balance, Gross Motor Function, and Daily Living Ability in Children With Cerebral Palsy: Systematic Review and Meta-analysis

**DOI:** 10.2196/38972

**Published:** 2022-11-09

**Authors:** Cong Liu, Xing Wang, Rao Chen, Jie Zhang

**Affiliations:** 1 School of Physical Education and Sport Training Shanghai University of Sport Shanghai China; 2 Department of Physical Education Donghua University Shanghai China

**Keywords:** virtual reality, cerebral palsy, balance, gross motor activities, activities of daily living, meta, motor, children, pediatrics

## Abstract

**Background:**

The increasing number of children with cerebral palsy (CP) has a serious impact on individuals, families, and society. As a new technology, virtual reality (VR) has been used in the rehabilitation of children with CP.

**Objective:**

This study aimed to systematically evaluate the effect of VR training on balance, gross motor function, and daily living ability in children with CP.

**Methods:**

PubMed, Embase, The Cochrane Library, Web of Science, and China National Knowledge Infrastructure databases were searched by computer, with the search period being from the establishment of each database to December 25, 2021, to collect randomized controlled trials (RCTs) on the effects of VR training on balance, gross motor function, and daily living ability in children with CP. The Cochrane risk of bias assessment tool was used to conduct quality assessment on the included literature, and RevMan software (version 5.3) was used to analyze data.

**Results:**

A total of 16 articles were included, involving 513 children with CP. VR training can improve the balance function (Pediatric Balance Scale: mean difference 2.06, 95% CI 1.15-2.97; *P*<.001; Berg Balance Scale: mean difference 3.66, 95% CI 0.29-7.02; *P*=.03) and gross motor function (standardized mean difference [SMD] 0.60, 95% CI 0.34-0.87; *P*<.001) of children with CP. However, there is still certain disagreement on the impact on daily living ability (SMD 0.37, 95% CI –0.04 to 0.78; *P*=.08); after removing the source literature with heterogeneity, VR training can improve the daily living ability of children with CP (SMD 0.55, 95% CI 0.30-0.81; *P*<.001).

**Conclusions:**

VR training can significantly improve the balance function and gross motor function of children with CP, but the effect on the daily living ability of children with CP remains controversial.

## Introduction

Cerebral palsy (CP) is a nonprogressive, persistent syndrome occurring in the brain of the fetus or infant [1]. The prevalence of CP is very high worldwide, and the prevalence can increase to 20-30 times in preterm or low–birth-weight newborns [2]. There are about 6 million children with CP in China, and the number is increasing at a rate of 45,000 per year [3]. Insufficiency of movement and abnormal posture are core symptoms of CP, and about 80% of children with CP have dyskinesia [4]. In addition, children with CP also have cognitive, communication, and perception-behavioral disorders; epilepsy; and other problems, which greatly limit their social participation and seriously affect their physical and mental health and quality of life.

Virtual reality (VR) refers to a virtual environment that is generated by a computer and can be interacted with. VR can mobilize the visual, auditory, tactile, and kinesthetic organs of children with CP, so that they can actively participate in the rehabilitation exercise. In this way, the central nerve conduction and peripheral motor control of children can be coordinated and unified, which is conducive to the rehabilitation of children [5]. Choi et al [6] conducted VR training for 40 children with CP for 4 weeks, 5 times a week, for 30 minutes each time. The study reported that compared with regular rehabilitation, the children in the VR group showed better results. Gagliardi et al [7] conducted a longitudinal study on 16 children with CP for 4 weeks, 5 times a week, for 30 minutes each time, and the results showed that the children’s walking ability and gross motor function improved. However, there was no significant change in daily living ability. In addition, a recent systematic review also showed that the combination of VR training and conventional rehabilitation training indicates better pure conventional rehabilitation training [8].

It has become a focus of scholars in China and abroad to improve the motor ability, abnormal posture, and quality of life of children with CP. Previous meta-analyses have shown that VR can significantly improve children’s hand function, balance function, gross motor function, and walking function [8-11]. The main problem this study intended to address is (1) whether VR training can significantly improve children’s balance and gross motor function after adding new evidence; (2) whether VR training can significantly improve children’s daily living ability; (3) whether the improvement of VR training on children with different types of CP is different; (4) whether the improvement of different types of VR on children with CP is different; and (5) whether different frequencies and periods of VR training have different improvement on children with CP. Address these problems will provide a basis for the formulation of accurate VR training program for children with CP.

## Methods

This study followed the requirements of the International Meta-analysis Writing Guidelines (The PRISMA [Preferred Reporting Items for Systematic Reviews and Meta-Analyses] statement for studies that evaluate health care interventions: explanation and elaboration) for the selection and use of methods.

### Literature Retrieval Strategy

In this study, 2 researchers independently retrieved 5 databases including PubMed, Embase, The Cochrane Library, Web of Science, and China National Knowledge Infrastructure (CNKI) to find randomized controlled trials (RCTs) of VR training effects on balance, gross motor function, and daily living ability in children with CP. Moreover, the sources of the CNKI database were limited to Chinese Social Sciences Citation Index and Chinese Science Citation Database, and the retrieval dates of all databases were from the establishment of each database to December 25, 2021. Supplementation measures were the tracking of relevant systematic reviews and references of the included literature. The retrieval adopted the method of combining subject words with free words—the Boolean operators “AND” and “OR” were used to combine and connect—and was determined after repeated prechecks. English search terms included “cerebral palsy,” “CP,” “children of cerebral palsy,” “cerebral palsy children,” “virtual reality,” “VR,” “virtual environment,” “video game,” “clinical trial,” “randomized controlled trial,” etc. An example of the literature retrieval strategy for the PubMed database is given (Multimedia Appendix 1).

### Inclusion and Exclusion Criteria

#### Inclusion Criteria

The inclusion criteria were as follows:

1. Population for research objects: clinically diagnosed children with spastic CP; their race and gender are not limited, and they are aged <16 years
2. Interventions for experimental group: VR training, VR training combined with conventional rehabilitation training, or VR training added on the basis of control group training
3. Comparison for control group interventions: daily physical activities, balance training, conventional rehabilitation training, or comprehensive rehabilitation training, etc
4. Outcome for outcome indicators: balance function was evaluated by Berg Balance Scale (BBS) and Pediatric Balance Scale (PBS); gross motor function was evaluated using the Gross Motor Function Measure Scale (GMFM), including GMFM-66, GMFM-E and GMFM-88; and the ability of daily living was assessed by Pediatric Evaluation of Disability Inventory (PEDI) and The Functional Independence Measure for Children (WeeFIM)
5. Study design for study type: RCTs

#### Exclusion Criteria

The exclusion criteria were as follows: non-RCTs; republished papers or papers with poor-quality evaluation; literature not in Chinese and English; full text not available; outcome indicators did not meet the requirements of this study or data could not be extracted; and intervention group content did not meet the requirements.

### Literature Screening and Data Extraction

In this study, 2 researchers independently retrieved literature from 5 databases and downloaded the retrieved literature into EndNote X9 software (Clarivate) in batches. After all the documents from the 5 databases were retrieved, duplicate documents were first removed in Endnote X9 software. Second, preliminary screening was carried out by reading the title and abstract. Third, the papers were screened according to the inclusion and exclusion criteria of this study. Finally, full-text readings were conducted to identify studies for final inclusion.

In all, 2 researchers extracted the data of the basic information and outcome indicators of the included papers and contacted the original author by email for unclear or missing data in the study. When the information extracted by 2 researchers was inconsistent, a third researcher would participate in a discussion to reach a consensus. The extracted information included basic information (author, year, country, sample size, type of CP, and patient age); experimental characteristics (intervention content, single intervention duration, frequency, and cycle); and outcome indicators. The researchers set up a table by reading the articles that met the inclusion criteria in detail and recording the relevant information.

### Quality Evaluation of Included Literature

This study used The Cochrane Collaboration’s Tool for Assessing Risk of Bias to analyze the literature using 7 methods: random sequence generation, allocation concealment, blinding of subjects and researchers, blinding of raters, incomplete outcome data, selective reporting, and other biases. These analysis results were categorized into 3 types of quality evaluation: low risk, unclear, and high risk. This process was carried out by 2 researchers independently. In case of disagreement, a third researcher would join in to discuss and make a decision. The quality of the literature was divided into 3 levels: grade A (low risk, meeting 4 or more items); grade B (low risk, meeting 2 or 3 items); and grade C (low risk, meeting 1 or no items, bias likely to occur) [12].

### Data Analysis

Data analysis was performed using RevMan software (version 5.3; Cochrane) and following the PRISMSA guidelines. The Q statistic test (*P* value) and *I*^2^ were used to test for heterogeneity. If there had been statistical heterogeneity between studies (*I*^2^>50%; *P*<.10), a random effects model would have been used for meta-analysis; otherwise, the fixed effects model would have been used. In this study, GMFM-66, GMFM-88, and GMFM-E were combined for gross motor function, and PEDI and WeeFIM were combined for daily living ability. Therefore, standardized mean difference (SMD) was used to calculate the indicators for gross motor function and daily living ability, and the mean difference (MD) was used to calculate the other indicators. There were no significant differences in the outcome variables between the groups in each comparison at baseline. At the end of the experiment, we chose scale scores of both the intervention group and the control group as the effect size, which reflects the intervention effect. Each effect size was given a point estimate and 95% CI. When *P*<.05, there was a significant difference between the intervention group and the control group, proving that the meta-analysis results were statistically significant. Publication bias testing was performed using Stata software (version 16.0; StataCorp) [13].

## Results

### Literature Retrieval Results

**Figure 1:**
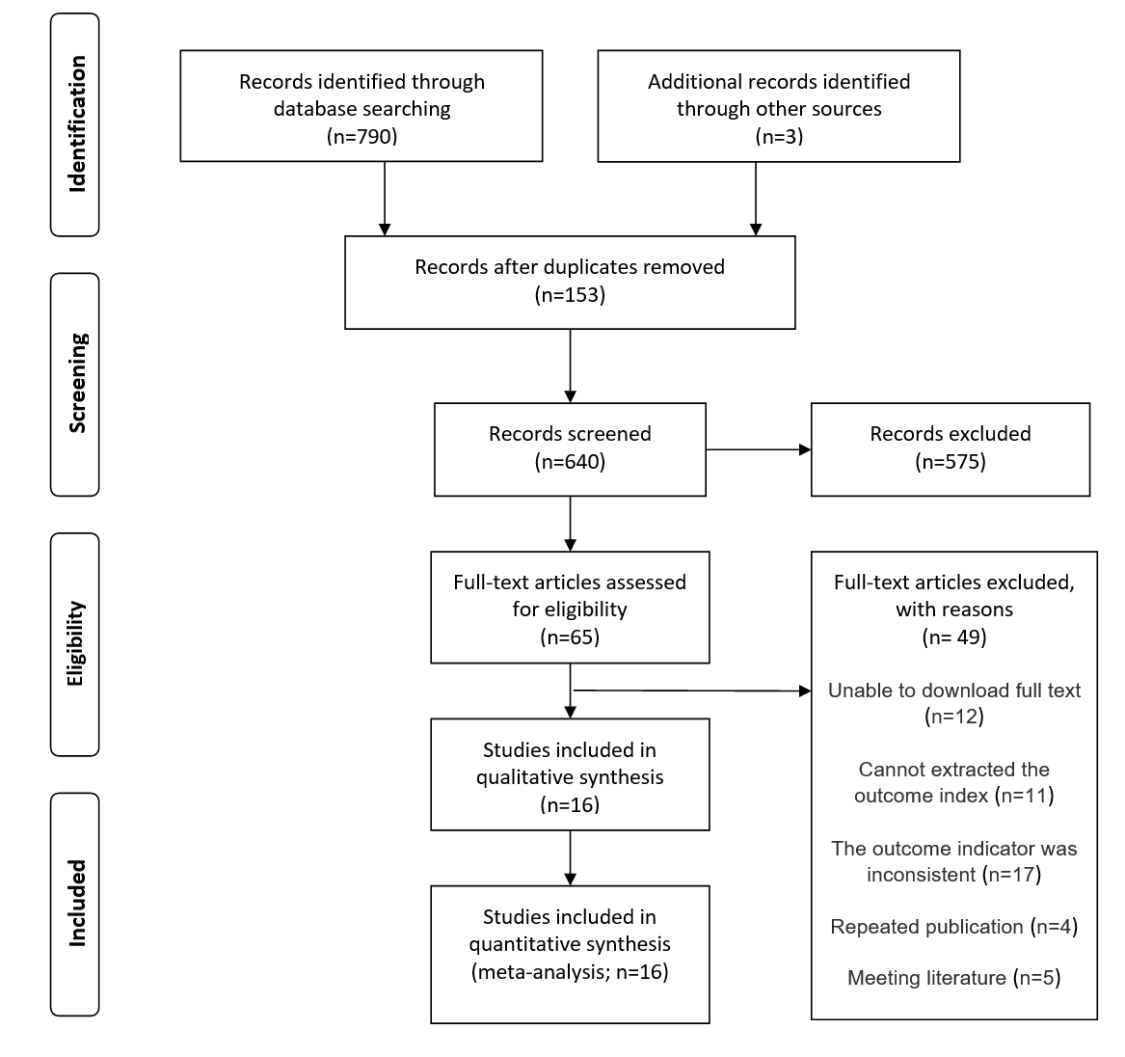
Literature screening process.

In all, 2 researchers searched 5 databases, including PubMed (n=82), Embase (n=191), The Cochrane Library (n=147), Web of Science (n=359), and CNKI (n=11). A total of 793 papers were retrieved, including 11 Chinese papers, 759 English papers, and 3 papers [14-16] from the previous systematic review of other people. After duplicate papers were excluded by Endnote X9 software, there were 640 papers left. Subsequently, 65 papers remained after primary screening, and lastly, 16 papers were left after the full-text rescreening [14-29]. As a result, 16 RCTs were finally included, as shown in Figure 1.

### Basic Features of Included Studies

A total of 16 articles representing16 RCTs were included—more specifically, 5 Chinese articles and 11 English articles. The publication period is from 2013 to 2021, and the countries of publication are China, Turkey, India, and South Korea. A total of 513 children with CP were included, and the CP types included spastic hemiplegia and spastic diplegia. The intervention of the experimental group included virtual time-limited training and the combination of VR training and regular rehabilitation training, etc; and the intervention of the control group included daily physical activities and regular rehabilitation training. The intervention duration of VR training was from 15-60 minutes, the frequency was from 2-6 times a week, and the cycle was from 3-12 weeks. (Multimedia Appendix 2).

### Quality Evaluation of the Included Literature

A total of 16 RCTs were included in this study, and their risk of bias was shown in Multimedia Appendix 3. All 16 RCTs described the generation of random sequences, among which 6 RCTs [14,17,19,21,23,24] described methods of allocation concealment, 6 RCTs [18-23] applied the blinding method for researchers and subjects, 6 RCTs [17,19-21,23,24] used the blinding method for raters, and all 16 RCTs had complete data and did not selectively report them.

### Meta-analysis Results

#### VR Effects on the Balance Function of Children With CP

**Figure 2:**
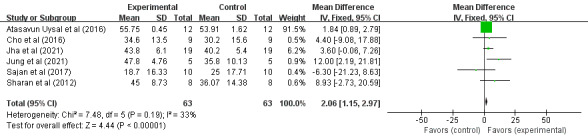
Forest plot of the effect of virtual reality on the Pediatric Balance Scale scores in children with cerebral palsy. IV: inverse variance.

A total of 126 cases of children with CP in 6 RCTs participated in the VR training and had relevant scoring conducted by using PBS evaluation, as shown in Figure 2. The results of the heterogeneity test (*I*^2^=33%; *P*=.19) indicated that there was no statistical heterogeneity among the studies, so the fixed effects model was used for analysis. Meta-analysis results showed that VR training was able to improve the PBS scores of children with CP (MD 2.06, 95% CI 1.15-2.97; *P*<.001), indicating that VR training could significantly improve the balance function of children with CP compared to the control group.

**Figure 3:**
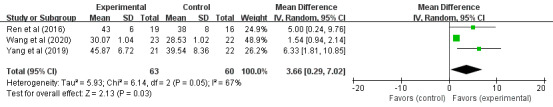
Forest plot of the effect of virtual reality on the Berg Balance Scale scores in children with cerebral palsy. IV: inverse variance.

A total of 123 cases of children with CP in 3 RCTs participated in the VR training and had relevant scoring conducted by using BBS evaluation, as shown in Figure 3. Heterogeneity test results (*I*^2^=67%; *P*=.05) indicated statistical heterogeneity between studies; therefore, the random effects model was used for analysis. Meta-analysis results showed that VR training was able to improve the BBS scores of children with CP (MD 3.66, 95% CI 0.29-7.02; *P*=.03), indicating that VR training was able to improve the balance function of children with CP significantly compared to the control group. Since only 3 papers were included in this analysis, so no sensitivity analysis was performed.

#### Influence of VR on the Gross Motor Function of Children With CP

**Figure 4:**
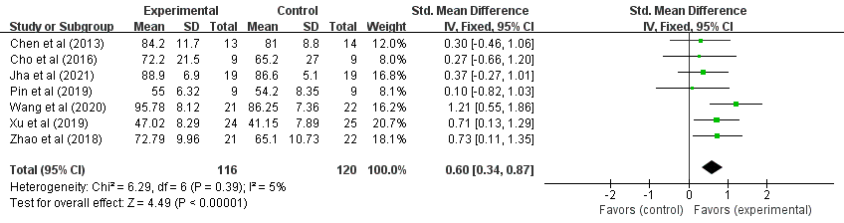
Forest plot of the effects of virtual reality on the gross motor function in children with cerebral palsy. IV: inverse variance.

A total of 236 cases of children with CP in 7 RCTs participated in VR training for the impact on their gross motor function, as shown in Figure 4. Heterogeneity test results (*I*^2^=5%; *P*=.39) indicated that there was no statistical heterogeneity between studies, so the fixed effects model was used for analysis. Meta-analysis results showed that VR training improved gross motor function in children with CP (SMD 0.60, 95% CI 0.34-0.87; *P*<.001), indicating that VR training could significantly improve gross motor function in children with CP compared to the control group.

A subgroup analysis in terms of CP type, training frequency, and period is shown in Table 1. The types of CP were divided into hemiplegia, diplegia, and other types, and the results showed that, compared to the control group, VR training only improved the gross motor function of children with hemiplegia significantly (*P*<.001). The training frequency was divided into ≤4 days/week and >4 days/week, and the results showed that, compared to the control group, VR training >4 days/week significantly improved the gross motor function of children with CP (*P*<.001). The training period was divided into <6 weeks and ≥6 weeks, and the results showed that, compared to the control group, both groups showed significant improvement on the gross motor function of children with CP (*P*<.001 and *P*=.002, respectively).

**Table 1:** Subgroup analysis of the effects of virtual reality training on gross motor function in children with cerebral palsy.

Group		Study, n	*I*^2^ (%)	Model	SMD^a^ (95% CI)	*P* value
**Type of cerebral palsy**						
	Hemiplegia	2	0	Fixed effects model	0.72 (0.30 to 1.14)	<.001
	Diplegia	2	0	Fixed effects model	0.28 (–0.24 to 0.81)	.29
	Other	3	52	Random effects model	0.64 (–0.00 to 1.29)	.05
**Training frequency**						
	≤4 days/week	4	0	Fixed effects model	0.29 (–0.11 to 0.68)	.15
	>4 days/week	3	0	Fixed effects model	0.86 (0.51 to 1.22)	<.001
**Training cycle**						
	<6 weeks	2	0	Fixed effects model	0.72 (0.30 to 1.14)	<.001
	≥6 weeks	5	31	Fixed effects model	0.53 (0.19 to 0.87)	.002

^a^SMD: standardized mean difference.

#### Effect of VR on the Daily Living Ability of Children With CP

**Figure 5:**
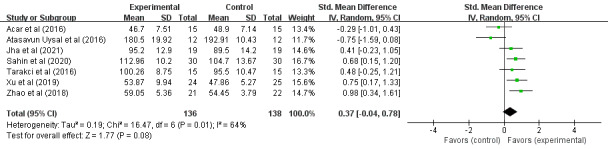
Forest plot of the effects of virtual reality on the daily living ability in children with cerebral palsy. IV: inverse variance.

A total of 274 children with CP from 7 RCTs participated in the research on the VR training effect on the daily living ability of CP children, as shown in Figure 5. The results of the heterogeneity test (*I*^2^=64%; *P*=.01) indicated that there was statistical heterogeneity among the studies, so the random effects model was used for analysis. Meta-analysis results showed that VR training did not improve the daily living ability of children with CP (SMD 0.37, 95% CI –0.04 to 0.78; *P*=.08), indicating that, compared to the control group, VR training had no effect on the daily living ability of children with CP. There was no advantage in improving the daily living ability of children with CP.

To explore sources of heterogeneity, a sensitivity analysis was performed by successive elimination of studies (see Table 2). After excluding Acar et al [14], there was no significant change in heterogeneity (SMD 0.45, 95% CI 0.07-0.89; *P*=.02), indicating that, compared to the control group, VR training had a significant effect on the daily living ability of children with CP. After excluding Atasavun Uysal et al [24] (*I*^2^=36%; *P*=.16), the fixed effects model was used for analysis (SMD 0.55, 95% CI 0.30-0.81; *P*=.001), indicating that, compared to the control group, the VR training significantly improved the daily living ability of children with CP. After excluding other studies, there was no significant change in heterogeneity, and the *P* values of the effect sizes were all greater than .05. Therefore, Atasavun Uysal et al [24] may be a source of heterogeneity.

**Figure 6:**
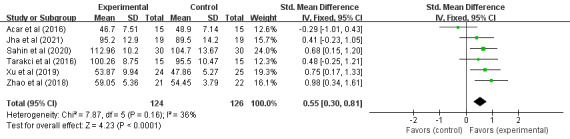
Forest plot of the effects of virtual reality on the daily living ability in children with cerebral palsy (excluding Atasavun Uysal et al [24]). IV: inverse variance.

After excluding Atasavun Uysal et al [24], a total of 250 cases of children with CP in 6 RCTs participated in VR training on their daily living ability, as shown in Figure 6. The results of the heterogeneity test (*I*^2^=36%; *P*=.16) indicated that there was no statistical heterogeneity among the studies, so the fixed effects model was adopted for analysis. Meta-analysis results showed that VR training significantly improved the daily living ability of children with CP (SMD 0.55, 95% CI 0.30-0.81; *P*<.001), indicating that, compared to the control group, VR training was able to significantly improve the daily living ability of children with CP.

A subgroup analysis of VR system type, training frequency, and training period is presented in Table 3. The types of VR systems were divided into Wii (Nintendo), Kinect (Microsoft), and other types. The results showed that, compared to the control group, only the Kinect system could significantly improve the daily living ability of children with CP (*P*<.001). Additionally, “other types” only had 1 study and is therefore not representative. The training frequency was divided into ≤4 days/week and >4 days/week, and the results showed that, compared to the control group, both groups significantly improved the daily activities of children with CP (*P*=.02 and *P*<.001, respectively). The training period was divided into ≤6 weeks and >6 weeks, and the results showed that, compared to the control group, VR training for >6 weeks significantly improved the daily activities of children with CP (*P*=.005).

**Table 2:** Combined effect of daily living ability after excluding individual studies.

Study eliminated	SMD^a^ (95% CI)	*P* value (combined effect)	*I*^2^ (%)	*P* value (heterogeneity)	Model
Acar et al [14], 2016	0.48 (0.07 to 0.89)	.02	58	.03	Random effects model
Atasavun Uysal et al [24], 2016	0.55 (0.30 to 0.81)	.001	36	.16	Fixed effects model
Jha et al [17], 2021	0.35 (–0.13 to 0.84)	.15	70	.006	Random effects model
Sahin et al [20], 2020	0.30 (–0.19 to (0.79)	.23	68	.009	Random effects model
Tarakci et al [23], 2016	0.34 (–0.13 to 0.82)	.16	70	.006	Random effects model
Xu et al [26], 2019	0.29 (–0.18 to 0.77)	.23	67	.01	Random effects model
Zhao et al [28], 2018	0.27 (–0.17 to 0.70)	.23	62	.02	Random effects model

^a^SMD: standardized mean difference.

**Table 3:** Subgroup analysis of the effects of virtual reality training on daily living ability in children with cerebral palsy.

Group			Study, n		*I*^2^ (%)		Model		SMD^a^ (95% CI)		*P* value
**Virtual reality system type**											
	Wii	2		54		Random effects model		0.09 (–0.66 to 0.85)		.81	
	Kinect	3		0		Fixed effects model		0.69 (0.35 to 1.03)		<.001	
	Other	1		N/A^b^		N/A		0.75 (0.17 to 1.33)		.01	
**Training frequency**											
	≤4 days/week	4		36		Fixed effects model		0.39 (0.07 to 0.70)		.02	
	>4 days/week	2		0		Fixed effects model		0.85 (0.42 to 1.28)		<.001	
**Training cycle**											
	≤6 weeks	4		60		Random effects model		0.48 (–0.03, to 1.00)		.06	
	>6 weeks	2		0		Fixed effects model		0.61 (0.19 to 1.03)		.005	

^a^SMD: standardized mean difference.

^b^N/A: not applicable.

### Testing of Publication Bias

Since the BBS indicators used in the study were limited, only the PBS, gross motor function, and daily living ability indicators were tested by Begg funnel plot. The test result of PBS was *Z*=0.75 (*P_r_*>|*z*|=.45); the test result of gross motor function was *Z*=0.9 (*P_r_*>|*z*|=.37); and the test result of daily living ability was *Z*=1.50 (*P_r_*>|*z*|=.13). Therefore, there was no publication bias in this study (Multimedia Appendix 4).

## Discussion

### Principal Findings

VR technology is a new practical technology; it integrates computer software and hardware, artificial intelligence, sensing, simulation, and other scientific technologies to give users an immersive sense and can provide users with the ability to interact with virtual objects. At present, VR technology has been widely used in the field of medicine to promote the rapid recovery of patients [20-32].

The balance function depends on the central nervous system in many aspects. In addition to the central nervous system injury, children with CP also have skeletal deformity, triceps spasm in the calf, increased muscle tension on one side, and clubfoot, which seriously affect the balance ability of children with CP [33,34]. VR training can enable children to actively complete hip flexion, abduction, and external rotation in a standing state, which is conducive to the improvement of balance function. VR training can also provide purposeful tasks and give sensory feedback, which is conducive to the recovery of neurological function— this in turn improves the balance function [35]. In this study, PBS and BBS were used to evaluate the balance function of children with CP. The results showed that VR training could significantly improve the balance function of children with CP compared to the control group, which are consistent with previous studies. Jaume-i-Capó et al [36] conducted VR game training for 9 adult patients with CP for 24 weeks, and the results showed that the balance function of the patients was significantly improved. Jelsma et al [37] conducted VR training (Nintendo Wii Fit) for 25 minutes, 4 times a week for 3 weeks on 14 children with CP, and the results showed that the balance and walking function of children with CP were significantly improved. Pourazar et al [38] conducted VR training for 10 children with CP for 20 times, and the results showed that the balance function of the experimental group was significantly improved compared to the control group.

VR training, which provides children with CP with a similar environment to the real world, gives a visual, auditory, and kinesthetic stimuli and interaction; activates the brain specific movement area; increases the blood flow of motor cortex; and prompts the cortical neural and cortex tissue improvement and restructuring. Therefore, the movement function of children can be compensated and activated, and motor function is improved [39]. In addition, VR training requires children to complete movements such as running and jumping to improve their motor function. In this study, GMFM was used to evaluate gross motor function in children with CP. The results showed that VR training was able to significantly improve gross motor function in children with CP compared to the control group. The findings are consistent with previous studies. Collange Grecco et al [40] performed VR training combined with continuous transcranial direct current stimulation on children with CP and found that gross motor function and gait were significantly improved. Burdea et al [41] conducted VR training for children with CP 3 times a week for 12 weeks and found that movement disorders in children with CP were significantly improved. In addition, a review also showed that VR training was beneficial to the improvement of gross motor function in children with CP [42]. The results of this study showed that VR training only significantly improved the gross motor function in children with hemiplegia but not in children with diplegia, which may be related to the limited studies involved.

In this study, WeeFIM and PEDI were used to evaluate the daily living ability of children with CP. The results showed that there was significant heterogeneity between studies and that VR training did not significantly improve the daily living ability of children with CP compared to the control group. To reduce the heterogeneity between studies, the method of successive elimination of studies was used for sensitivity analysis, and the results showed that the heterogeneity came from Atasavun Uysal et al [24]. After reading the full text of the paper again, it was speculated that the main reason was that the PEDI scoring method was using original scores instead of normative scores, which led to excessive heterogeneity among studies. After removing and combining the effect size, it was found that compared to the control group, the VR training group significantly improved the children’s daily living ability, which was consistent with previous studies. Tarakci et al [23] conducted VR training twice a week for 20 minutes each time, for 12 weeks on 15 children with CP, and the results showed that compared to the control group, the children in the VR training group had significantly improved their daily living ability. You et al [43] conducted VR training on children with CP and found that the cerebral cortex related to self-feeding and dressing was improved. VR training may improve the daily living ability of children with CP for the following reasons: the virtual environment provided by VR training can be highly consistent with the real world, which is conducive to mobilizing all organs of children with CP and transferring skills acquired by children with CP to daily life; and VR training can provide a variety of sensory stimulation and feedback information, which is more novel than traditional rehabilitation. Therefore, children are more actively involved in rehabilitation training with higher compliance, which makes the connection of peripheral-central-peripheral nerve conduction pathways more frequent. All these improvements are beneficial to the rehabilitation of children with CP [44,45]. This study shows that the Kinect system has a more obvious improvement effect on children’s daily living ability, whereas the Wii system has no obvious improvement effect on children’s daily living ability. This finding may be due to the fact that the Kinect system more enriched than the Wii system, its sensory stimulation is more obvious, and the human-computer interaction is more natural and convenient. In addition, the results of this study showed that the longer the training cycle, the better the improvement of gross motor function and daily living ability of children with CP. Therefore, in future studies, the rehabilitation training time of children with CP should be extended.

Our study found that VR can improve balance function and gross motor function in children with CP, which is consistent with previous studies. After removing the literature with high heterogeneity [24], the results showed that VR can improve the daily living ability of children, which is also similar to previous studies. In addition, our study also found that the type of VR system, the type of CP, and the training time can all affect the rehabilitation of children.

### Limitations

This study only included Chinese- and English-language literature; the indicators included were limited to GMFM, BBS, and PBS; and the number of studies included in several groups was relatively small in the subgroup analysis, so the conclusions obtained have certain limitations. The content of VR training is diverse; the content of regular rehabilitation is not consistent; and the duration, frequency, and cycle of training are also different, which may also be the reason for the heterogeneity of studies. Part of the literature included in this study did not clarify whether allocation concealment was used, the researchers or subjects were blinded, or the evaluators were blinded, so there may be bias in the research results.

### Conclusions

Meta-analysis results showed that compared to the control group, VR training significantly improves the balance function and gross motor function of children with CP, but the impact on the social function of children with CP is still controversial. Therefore, more RCTs with high quantity and quality are suggested to be performed in the future in an effort to further confirm the treatment effects of VR training on balance function, gross motor function, and the daily living ability among children with CP, as well as to offer more solid evidence for clinical trials. Therefore, we suggest carrying out more high-quality RCTs with large samples in the future to further confirm the efficacy of VR training on balance ability, gross motor function, and the daily living ability of children with CP and provide more reliable evidence for clinical practice.

## Appendix

Multimedia Appendix 1 Literature retrieval strategy for the PubMed database.

Multimedia Appendix 2 Basic features of the included papers.

Multimedia Appendix 3 Risk of bias of the included papers.

Multimedia Appendix 4 Begg funnel plots.

## Abbreviations

BBS: Berg Balance Scale
CNKI: China National Knowledge Infrastructure
CP: cerebral palsy
GMFM: Gross Motor Function Measure Scale
MD: mean difference
PBS: Pediatric Balance Scale
PEDI: Pediatric Evaluation of Disability Inventory
PRISMA: Preferred Reporting Items for Systematic Reviews and Meta-Analyses
RCT: randomized controlled trial
SMD: standardized mean difference
VR: virtual reality
WeeFIM: The Functional Independence Measure for Children

## References

[1] Koman LA, Smith BP, Shilt JS (2004). Cerebral palsy. Lancet.

[2] Oskoui M, Coutinho F, Dykeman J, Jetté Nathalie, Pringsheim T (2013). An update on the prevalence of cerebral palsy: a systematic review and meta-analysis. Dev Med Child Neurol.

[3] Zelnik N, Lahat E, Heyman E, Livne A, Schertz M, Sagie L, Fattal-Valevski A (2016). The role of prematurity in patients with hemiplegic cerebral palsy. J Child Neurol.

[4] Brandenburg JE, Fogarty MJ, Sieck GC (2019). A critical evaluation of current concepts in cerebral palsy. Physiology (Bethesda).

[5] Kelly C, Foxe JJ, Garavan H (2006). Patterns of normal human brain plasticity after practice and their implications for neurorehabilitation. Arch Phys Med Rehabil.

[6] Choi JY, Yi S, Ao L, Tang X, Xu X, Shim D, Yoo B, Park ES, Rha D (2021). Virtual reality rehabilitation in children with brain injury: a randomized controlled trial. Dev Med Child Neurol.

[7] Gagliardi C, Turconi AC, Biffi E, Maghini C, Marelli A, Cesareo A, Diella E, Panzeri D (2018). Immersive virtual reality to improve walking abilities in cerebral palsy: a pilot study. Ann Biomed Eng.

[8] Fandim JV, Saragiotto BT, Porfírio Gustavo José Martiniano, Santana RF (2021). Effectiveness of virtual reality in children and young adults with cerebral palsy: a systematic review of randomized controlled trial. Braz J Phys Ther.

[9] Wu J, Loprinzi PD, Ren Z (2019). The rehabilitative effects of virtual reality games on balance performance among children with cerebral palsy: a meta-analysis of randomized controlled trials. Int J Environ Res Public Health.

[10] Chen Y, Fanchiang HD, Howard A (2018). Effectiveness of virtual reality in children with cerebral palsy: a systematic review and meta-analysis of randomized controlled trials. Phys Ther.

[11] Chen Y, Lee SY, Howard AM (2014). Effect of virtual reality on upper extremity function in children with cerebral palsy: a meta-analysis. Pediatr Phys Ther.

[12] Wu Z, Wang Z, Songyan L (2017). Meta-analysis of Chinese obese adolescents weight-losing effect by exercise. Article in Chinese. Journal of Shenyang Sport University.

[13] Fernández-Rodríguez Rubén, Álvarez-Bueno Celia, Martínez-Ortega Isabel A, Martínez-Vizcaíno Vicente, Mesas AE, Notario-Pacheco B (2022). Immediate effect of high-intensity exercise on brain-derived neurotrophic factor in healthy young adults: a systematic review and meta-analysis. J Sport Health Sci.

[14] Acar G, Altun GP, Yurdalan S, Polat MG (2016). Efficacy of neurodevelopmental treatment combined with the Nintendo(®) Wii in patients with cerebral palsy. J Phys Ther Sci.

[15] Chen CL, Chen CY, Liaw MY, Chung CY, Wang CJ, Hong WH (2013). Efficacy of home-based virtual cycling training on bone mineral density in ambulatory children with cerebral palsy. Osteoporos Int.

[16] Cho C, Hwang W, Hwang S, Chung Y (2016). Treadmill training with virtual reality improves gait, balance, and muscle strength in children with cerebral palsy. Tohoku J Exp Med.

[17] Jha KK, Karunanithi GB, Sahana A, Karthikbabu S (2021). Randomised trial of virtual reality gaming and physiotherapy on balance, gross motor performance and daily functions among children with bilateral spastic cerebral palsy. Somatosens Mot Res.

[18] Jung S, Song S, Lee D, Lee K, Lee G (2021). Effects of Kinect video game training on lower extremity motor function, balance, and gait in adolescents with spastic diplegia cerebral palsy: a pilot randomized controlled trial. Dev Neurorehabil.

[19] Pin TW, Butler PB (2019). The effect of interactive computer play on balance and functional abilities in children with moderate cerebral palsy: a pilot randomized study. Clin Rehabil.

[20] Şahin S, Köse Barkın, Aran OT, Bahadır Ağce Z, Kayıhan H (2020). The effects of virtual reality on motor functions and daily life activities in unilateral spastic cerebral palsy: a single-blind randomized controlled trial. Games Health J.

[21] Sajan JE, John JA, Grace P, Sabu Sneha Sara, Tharion George (2017). Wii-based interactive video games as a supplement to conventional therapy for rehabilitation of children with cerebral palsy: a pilot, randomized controlled trial. Dev Neurorehabil.

[22] Sharan D, Ajeesh PS, Rameshkumar R, Mathankumar M, Paulina R Jospin, Manjula M (2012). Virtual reality based therapy for post operative rehabilitation of children with cerebral palsy. Work.

[23] Tarakci D, Ersoz Huseyinsinoglu B, Tarakci E, Razak Ozdincler A (2016). Effects of Nintendo Wii-Fit video games on balance in children with mild cerebral palsy. Pediatr Int.

[24] Atasavun Uysal S, Baltaci G (2016). Effects of Nintendo Wii Training on occupational performance, balance, and daily living activities in children with spastic hemiplegic cerebral palsy: a single-blind and randomized trial. Games Health J.

[25] Ren Kai, Gong Xiao-Ming, Zhang Rong, Chen Xiu-Hui (2016). Effects of virtual reality training on limb movement in children with spastic diplegia cerebral palsy. Article in Chinese. Zhongguo Dang Dai Er Ke Za Zhi.

[26] Xu Y, Zhao XK, Chen MY, Zhu M, Du SJ, Zhang L, Xuan XY (2019). Effect of virtual reality on therapeutic pain in children with spastic cerebral palsy. Article in Chinese. Chinese Journal of Rehabilitation Theory and Practice.

[27] Wang Y, Ma X, An H, Shi Y, Luo C, Song J, Qian X, Yin L (2020). Effect of virtual reality technology on balance function and gross motor function in children with spastic cerebral palsy. Article in Chinese. Chinese Nursing Management.

[28] Zhao X, Zhang Y, Tang J, Wang C, Zhang L, Zhu M, Li H, Du S (2018). The effect of combining constraint-induced movement therapy with virtual reality games in rehabilitating the motor function of hemiplegic children with cerebral palsy. Article in Chinese. Chin J Phys Med Rehabil.

[29] Yang MX, Zhang WT, Fu JM, Gu XD (2019). Effect of virtual reality training on balance function in children with spastic cerebral palsy. Article in Chinese. Chin J Phys Med Rehabil.

[30] Garcia LM, Birckhead BJ, Krishnamurthy P, Sackman J, Mackey IG, Louis RG, Salmasi V, Maddox T, Darnall BD (2021). An 8-week self-administered at-home behavioral skills-based virtual reality program for chronic low back pain: double-blind, randomized, placebo-controlled trial conducted during COVID-19. J Med Internet Res.

[31] Ridout B, Kelson J, Campbell A, Steinbeck K (2021). Effectiveness of virtual reality interventions for adolescent patients in hospital settings: systematic review. J Med Internet Res.

[32] Wu J, Zeng A, Chen Z, Wei Y, Huang K, Chen J, Ren Z (2021). Effects of virtual reality training on upper limb function and balance in stroke patients: systematic review and meta-meta-analysis. J Med Internet Res.

[33] Bolton DAE, Brown KE, McIlroy William E, Staines W Richard (2012). Transient inhibition of the dorsolateral prefrontal cortex disrupts somatosensory modulation during standing balance as measured by electroencephalography. Neuroreport.

[34] Aisen ML, Kerkovich D, Mast J, Mulroy S, Wren TA, Kay RM, Rethlefsen SA (2011). Cerebral palsy: clinical care and neurological rehabilitation. Lancet Neurol.

[35] Lange BS, Requejo P, Flynn SM, Rizzo A, Valero-Cuevas F, Baker L, Winstein C (2010). The potential of virtual reality and gaming to assist successful aging with disability. Phys Med Rehabil Clin N Am.

[36] Jaume-i-Capó Antoni, Martínez-Bueso Pau, Moyà-Alcover Biel, Varona J (2014). Interactive rehabilitation system for improvement of balance therapies in people with cerebral palsy. IEEE Trans Neural Syst Rehabil Eng.

[37] Jelsma J, Pronk M, Ferguson G, Jelsma-Smit Dorothee (2013). The effect of the Nintendo Wii Fit on balance control and gross motor function of children with spastic hemiplegic cerebral palsy. Dev Neurorehabil.

[38] Pourazar M, Bagherzadeh F, Mirakhori F (2021). Virtual reality training improves dynamic balance in children with cerebral palsy. Int J Dev Disabil.

[39] Karim H, Schmidt B, Dart D, Beluk N, Huppert T (2012). Functional near-infrared spectroscopy (fNIRS) of brain function during active balancing using a video game system. Gait Posture.

[40] Collange Grecco LA, de Almeida Carvalho Duarte N, Mendonça Mariana E, Galli M, Fregni F, Oliveira CS (2015). Effects of anodal transcranial direct current stimulation combined with virtual reality for improving gait in children with spastic diparetic cerebral palsy: a pilot, randomized, controlled, double-blind, clinical trial. Clin Rehabil.

[41] Burdea GC, Cioi D, Kale A, Janes WE, Ross SA, Engsberg JR (2013). Robotics and gaming to improve ankle strength, motor control, and function in children with cerebral palsy--a case study series. IEEE Trans Neural Syst Rehabil Eng.

[42] Ren Z, Wu J (2019). The effect of virtual reality games on the gross motor skills of children with cerebral palsy: a meta-analysis of randomized controlled trials. Int J Environ Res Public Health.

[43] You SH, Jang SH, Kim Y, Kwon Y, Barrow I, Hallett M (2005). Cortical reorganization induced by virtual reality therapy in a child with hemiparetic cerebral palsy. Dev Med Child Neurol.

[44] Walker ML, Ringleb SI, Maihafer GC, Walker R, Crouch JR, Van Lunen B, Morrison S (2010). Virtual reality-enhanced partial body weight-supported treadmill training poststroke: feasibility and effectiveness in 6 subjects. Arch Phys Med Rehabil.

[45] Turolla A, Dam M, Ventura L, Tonin P, Agostini M, Zucconi C, Kiper P, Cagnin A, Piron L (2013). Virtual reality for the rehabilitation of the upper limb motor function after stroke: a prospective controlled trial. J Neuroeng Rehabil.

