# Supplementary material for: The Effects of Virtual Reality Training on Balance, Gross Motor Function, and Daily Living Ability in Children With Cerebral Palsy: Systematic Review and Meta-analysis
Source: JMIR Serious Games. 2022 Nov 9;10(4):e38972. doi: 10.2196/38972 (PMC9685515; doi:10.2196/38972)
Supplement: Multimedia Appendix 1 [file games_v10i4e38972_app1.pdf]

#1 Virtual Reality[Mesh]

#2VR[Title/Abstract] OR virtual environment[Title/Abstract] OR video game[Title/Abstract]

#3 #1 OR #2

#4 Cerebral Palsy[Mesh]

#5 CP[Title/Abstract] OR children of cerebral palsy[Title/Abstract] OR cerebral palsy children[Title/Abstract]

#6 #4 OR #5

#7 Randomized controlled trial[Publication Type]

#8 clinical trial[Publication Type]

#9Randomized[Title/Abstract] controlled[Title/Abstract] trial[Title/Abstract] clinical[Title/Abstract]

#10 #7 OR #8 OR #9

#3 AND #6 AND #10
