# Supplementary material for: The Effects of Virtual Reality Training on Balance, Gross Motor Function, and Daily Living Ability in Children With Cerebral Palsy: Systematic Review and Meta-analysis
Source: JMIR Serious Games. 2022 Nov 9;10(4):e38972. doi: 10.2196/38972 (PMC9685515; doi:10.2196/38972)
Supplement: Multimedia Appendix 2 [file games_v10i4e38972_app2.docx]

Table 1: Basic Features of the Included Papers

| Author/Year | Country | Age （E/C） | Sample size（E/C） | CP Type | Intervention  （E/C） | Dosage | Outcome  Indicators |
| --- | --- | --- | --- | --- | --- | --- | --- |
| Atasavun Uysal2016 | Turkey | 9.13±2.57/10.11±2.62Year | 12/12 | Spastic  Hemiplegic | E: Nintendo Wii Sports Games + traditional physiotherapy program； C: traditional physiotherapy program | 30min×2d×12wk | ①② |
| Tarakci2016 | Turkey | 10.46±2.69/10.53±2.79Year | 15/15 | Mixed | E：Nintendo Wii-Fit balance-based video games＋NDT ；C；conventional balance training＋NDT | 20min×2d×12wk | ③ |
| Sharan2012 | India | 8.88±3.23/10.38±4.41Year | 14/15 | Not specified | E：Nintendo Wii sports and Wii fit＋conventional rehabilitation modalities ；C：conventional rehabilitation modalities | -min×3d×3wk | ② |
| Sajan2017 | India | 10.6±3.78/12.4±4.93Year | 10/10 | Mixed | E: Nintendo Wii games＋conventional therapy;C：conventional t-herapy | 45min×3d×6wk | ② |
| Sahin2020 | Turkey | 10.5±3.62/10.06±3.24Year | 30/30 | Spastic  Hemiplegic | E：VR intervention（Kinect）＋Traditional Occupational Therapy；C：Traditional Occupational Therapy | 45min×2d×8wk | ③ |
| Pin2019 | Hong Kong | 8.92±2.25/9.59±1.87Year | 9/9 | Bilateral  Spastic | E：Interactive computer play training（TYROMOTION GmbH）＋usual physiotherapy programme；C：usual physiotherapy programme | 20min×4d×6wk | ④ |
| Jung2021 | Korea | 12.8±1.6/12±2.53Year | 5/5 | Spastic  Diplegia | E：Kinetic video game＋conventi-onal therapy;  C：conventional therapy | 40min×3d×6wk | ② |
| Jha2021 | India | 8.94±1.92/8.72±1.68Year | 19/19 | Bilateral  Spastic | E：Kinect-based virtual reality gaming＋physiotherapy；  C：physiotherapy | 30min×4d×6wk | ②③⑤ |
| Ren2016 | China | 4.75±0.83/4.5±1.17Year | 19/16 | Bilateral  Spastic | E:VR（Q4）＋ occupational therapy；  C：occupational therapy＋Physical therapy | 40min×5d×12wk | ⑤⑥ |
| Wang2020 | China | 7.42±2.39/7.21±2.16Year | 21/22 | Mixed | E：Kinect games ＋ Routine rehabilitation training；  C：Routine rehabilitation training | 40min×5d×12wk | ⑤⑥ |
| Xu2019 | China | 3.31±0.91/3.04±0.87Year | 24/25 | Spastic  Hemiplegic | E：VR＋Routine rehabilitation training；  C：Routine rehabilitation training | -min×5d×3wk | ①⑦ |
| Yang2019 | China | 4.63±1.23/4.57±1.17Year | 23/22 | Bilateral  Spastic | E：Biomaster VR＋balance＋Routine rehabilitation training；  C：balance＋Routine rehabilitation training | 20min×5d×12wk | ⑥ |
| Zhao2018 | China | 3.93±0.71/3.46±1.17Year | 25/25 | Spastic  Hemiplegic | E：Kinect＋Routine rehabilitation training＋CIMT；  C：Routine rehabilitation training＋CIMT | 60min×5d×3wk | ①⑤ |
| Acar2016 | Turkey | 9.53±3.04/9.73±2.86Year | 15/15 | Spastic  Hemiplegic | E：neurodevelopmental treatment＋Nintendo Wii and Wii Fit (Wii)；C：neurodevelopmental treatment | 15min×2d×6wk | ③ |
| Chen2013 | China | 8.7±2.1/8.6±2.2Year | 13/14 | Mixed | E：virtual cycling training（Eloton SimCycle Virtual Cycling System）；C：general physical activity | 40min×3d×12wk | ④ |
| Cho2016 | Korea | 10.2±3.4/9.4±3.8Year | 9/9 | Not  specified | E：virtual reality treadmill training（Nintendo Wii）＋general physical therapy；C：treadmill gait training＋general physical therapy | 30min×3d×8wk | ②⑤ |

Note:

NDT: Neuro-developmental treatment approach;

CIMT：Constraint-Induced Movement Therapy;

E: Experimental Group;

C：Control group;

①PEDI；②PBS；③WeeFIM；④GMFM-66；⑤GMFM-88；⑥BBS；⑦GMFM-E
