# Supplementary figures and images for: The Effects of Virtual Reality Training on Balance, Gross Motor Function, and Daily Living Ability in Children With Cerebral Palsy: Systematic Review and Meta-analysis
Source: JMIR Serious Games. 2022 Nov 9;10(4):e38972. doi: 10.2196/38972 (PMC9685515; doi:10.2196/38972)

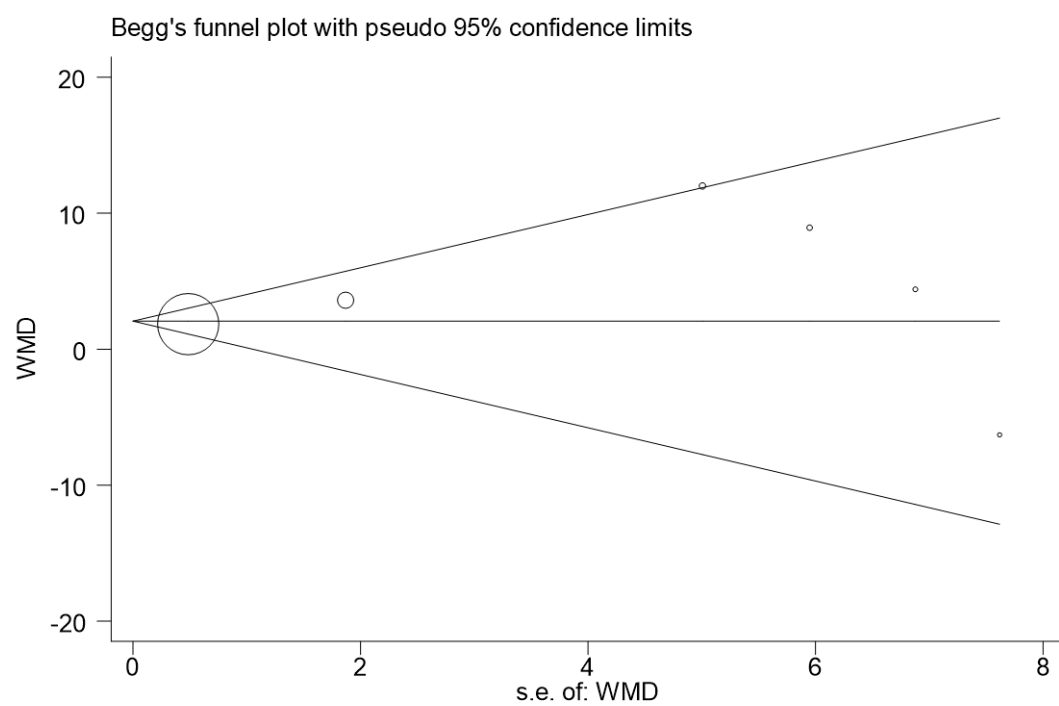

Figure S1

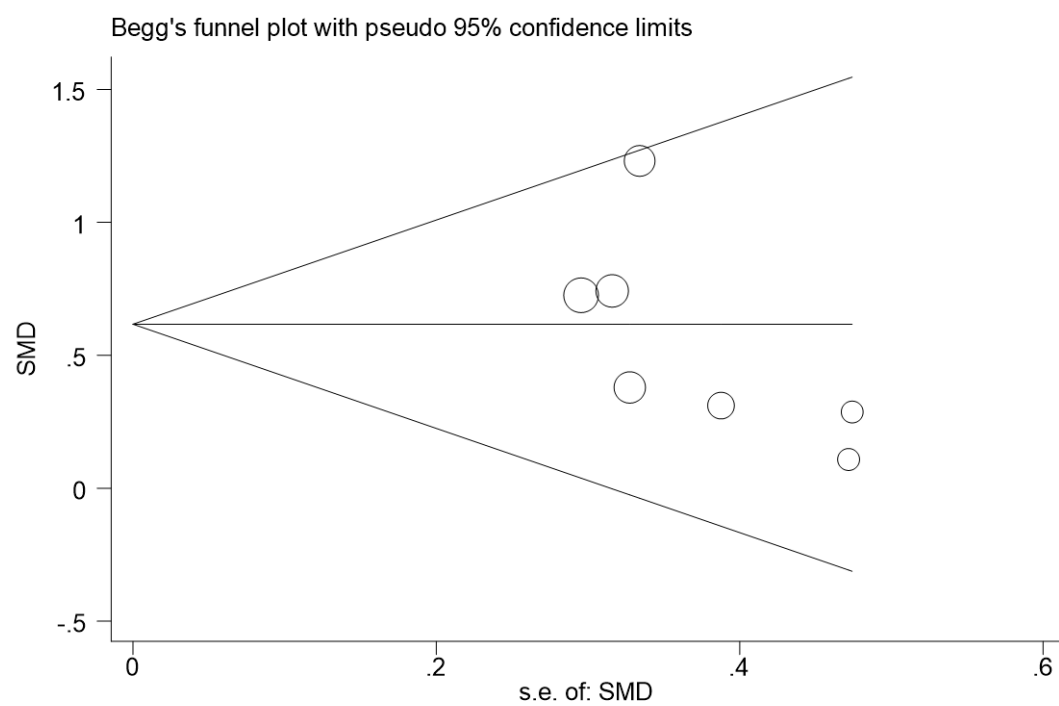

Figure S2

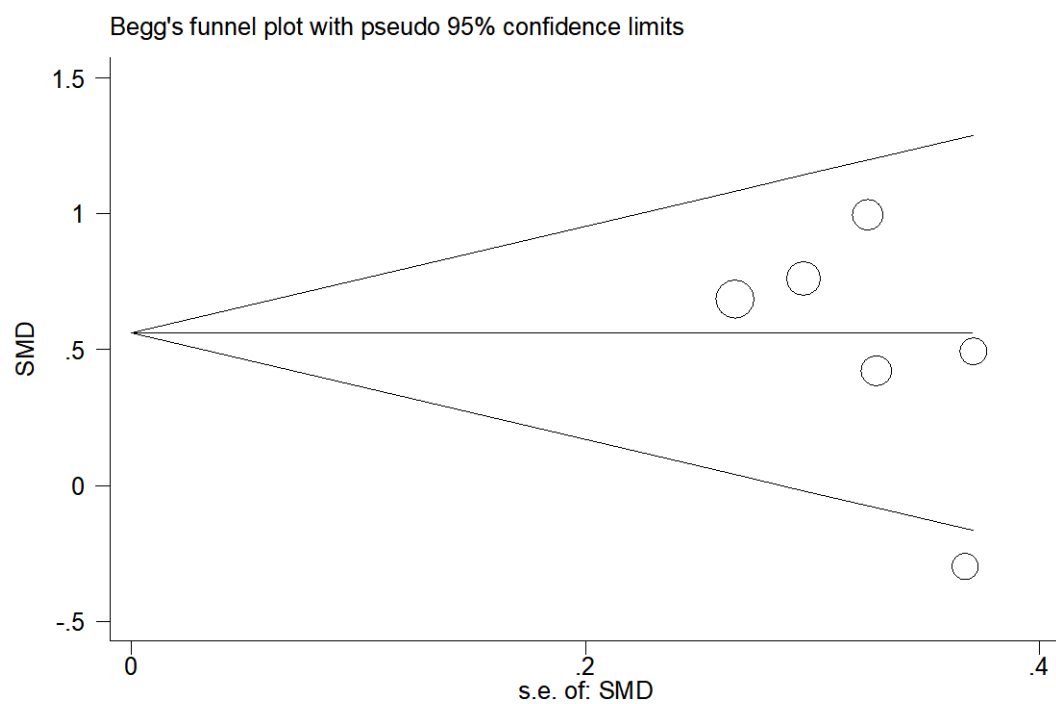

Figure S3

Supplement: Multimedia Appendix 4 [file games_v10i4e38972_app4.pdf]
