# Supplementary material for: The Effects of Virtual Reality Training on Balance, Gross Motor Function, and Daily Living Ability in Children With Cerebral Palsy: Systematic Review and Meta-analysis
Source: JMIR Serious Games. 2022 Nov 9;10(4):e38972. doi: 10.2196/38972 (PMC9685515; doi:10.2196/38972)
Supplement: Multimedia Appendix 3 [file games_v10i4e38972_app3.pdf]

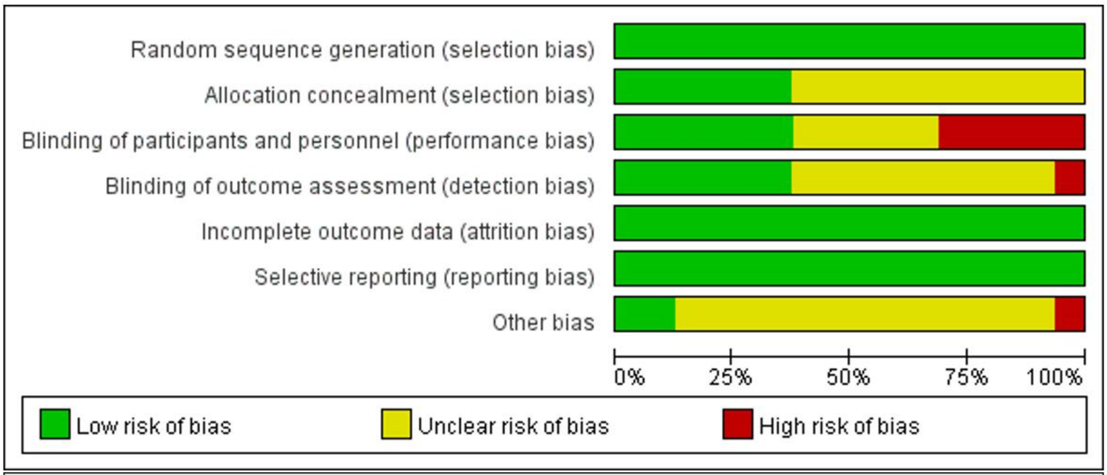
Random sequence generation (selection bias)
Allocation concealment (selection bias)
Blinding of participants and personnel (performance bias)
Blinding of outcome assessment (detection bias)
Incomplete outcome data (attrition bias)
Selective reporting (reporting bias)
Other bias
0%
25%
50%
75%
100%
Low risk of bias
Unclear risk of bias
High risk of bias

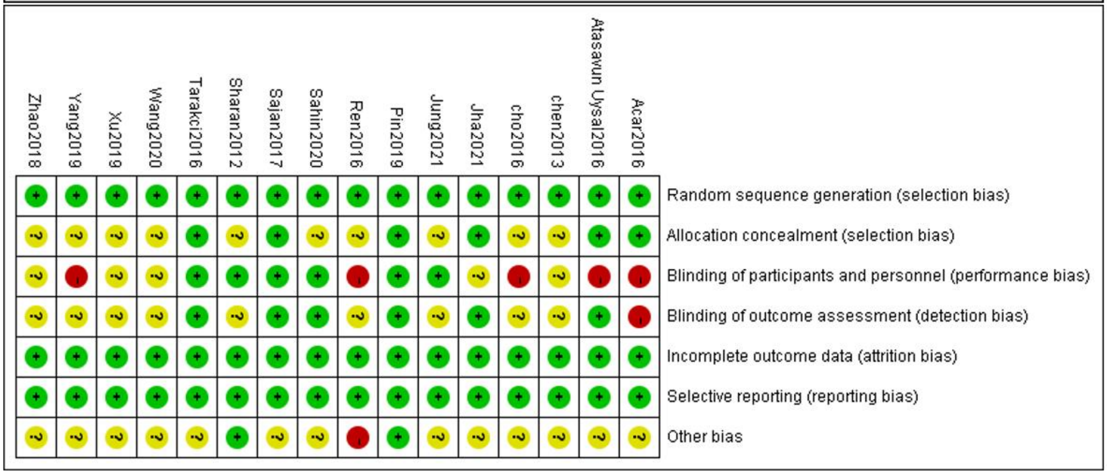
Zhao2018
Yang2019
Xu2019
Wang2020
Tarakci2016
Sharan2012
Sajan2017
Sahin2020
Ren2016
Pin2019
Jung2021
Jha2021
cho2016
chen2013
Atasavun Uysal2016
Acar2016
Random sequence generation (selection bias)
Allocation concealment (selection bias)
Blinding of participants and personnel (performance bias)
Blinding of outcome assessment (detection bias)
Incomplete outcome data (attrition bias)
Selective reporting (reporting bias)
Other bias
